# Development and Validation of a Machine Learning-Based Prediction Model for Illness Uncertainty in Patients with Malignant Tumors

**DOI:** 10.3390/healthcare14142160

**Published:** 2026-07-17

**Authors:** Yan Lu, Hui Zeng, Li Mao, Jingjing Gong, Yuxuan Wei, Xiaodan Li

**Affiliations:** 1 Department of Obstetrics and Gynecology, Peking University People’s Hospital, Beijing 100044, China; luyan@pkuph.edu.cn (Y.L.);; 2 College of Nursing, Hebei University, Baoding 071002, China

**Keywords:** illness uncertainty, malignant tumors, nomogram, anxiety, depression

## Abstract

**Highlights:**

**What are the main findings?**

- Anxiety was the only predictor shared by LASSO, Random Forest, and XGBoost. Seven consensus predictors supported by at least two algorithms were used to construct the nomogram.
- The nomogram showed good internal discrimination (AUC = 0.724) but exhibited calibration bias in time-stratified temporal validation (*p* < 0.05), revealing temporal heterogeneity.

**What are the implications of the main findings?**

- The model shifts illness uncertainty identification from post hoc assessment to admission screening, enabling proactive risk stratification in clinical practice.
- Modifiable predictors (anxiety, depression, social support, coping styles) offer clear targets for early psychosocial interventions.

**Abstract:**

**Objectives**: To develop and validate an individualized prediction model for assessing the risk of illness uncertainty in patients with malignant tumors, using a cross-sectional design. **Methods**: Patients with malignant tumors treated at Peking University People’s Hospital from August 2024 to January 2025 were enrolled. The Mishel Uncertainty in Illness Scale (MUIS) was used to classify patients into high-risk and low-to-moderate risk groups. Patients were divided into a model development set and a time-stratified validation set. The development set was further randomly split into a training set and an internal validation set at a 7:3 ratio. Three machine learning algorithms Least Absolute Shrinkage and Selection Operator (LASSO), Random Forest (RF), and eXtreme Gradient Boosting (XGBoost) were employed to screen for common predictors, and a nomogram was constructed based on logistic regression. The model’s performance was evaluated using the Area Under the Receiver Operating Characteristic Curve, calibration curves, and the Hosmer–Lemeshow test. **Results**: A total of 966 patients were included, with 676 in the development set and 290 in the time-stratified validation set. Seven predictors were ultimately identified for the nomogram: age, education level, diagnosis, depression, anxiety, medical coping modes, and social support. Notably, anxiety was the only variable jointly identified by all three algorithms, while the other six were commonly selected by both Random Forest and XGBoost. The nomogram showed good discrimination in training (AUC = 0.763, 95% CI: 0.717–0.808) and internal validation (AUC = 0.724, 95% CI: 0.645–0.803), with well-calibrated probabilities (*p* > 0.05). In time-stratified validation, discrimination was acceptable (AUC = 0.663, 95% CI: 0.58–0.739), but calibration revealed risk underestimation for low-to-moderate risk group (*p* < 0.05). **Conclusions**: The nomogram shows acceptable internal performance as an exploratory concurrent classifier rather than a genuine predictor, but calibration bias and modest temporal discrimination (AUC = 0.663) indicate it is not ready for clinical use. Further optimization and multi-center prospective validation are required.

## 1. Introduction

Illness uncertainty refers to a patients’ inability to determine the meaning of illness-related events and accurately predict the disease outcome [1]. This phenomenon is prevalent among patients with malignant tumors, with an incidence rate as high as 30–50% [2]. With continuous improvements in early diagnosis and treatment methods, the survival period of these patients has significantly prolonged [3]. However, as a persistent psychological stressor, illness uncertainty severely impacts their treatment compliance and quality of life [4]. Extensive research shows that illness uncertainty not only has a reciprocal causal relationship with negative emotions like anxiety and depression but may also affect tumor progression and long-term survival through neuroendocrine and immune pathways [5,6]. Therefore, early identification of patients with a high risk of illness uncertainty and the implementation of stratified psychological interventions are of great clinical significance for improving their overall prognosis.

Currently, clinical assessment of illness uncertainty primarily relies on the Mishel Uncertainty in Illness Scale [7], which comprises 33 items and requires approximately 10–15 min to complete. While brief versions such as the 22-item MUIS-C have been developed to reduce respondent burden [8], these tools still serve as direct measures of how patients perceive uncertainty, rather than as predictors of its occurrence [9]. Our model is not intended to replace the MUIS as a diagnostic tool. Instead, it offers a complementary approach to risk stratification, shifting the focus from direct measurement to predictive screening. The model draws on data that are either routinely available in clinical records or readily collectible via brief, validated questionnaires, thereby moving illness uncertainty identification from a reactive, post hoc exercise to a proactive screening process at admission. Of note, this does not mean that questionnaire-based data collection can be completely avoided; rather, it repurposes such data for early risk prediction. The predictive model developed in this study, through further external validation and implementation research, could be assessed for its feasibility and performance in real-world clinical practice.

In recent years, risk prediction models have been widely applied in the management of psychological complications in patients with tumors [10,11]. Based on common risk factors, these models use statistical methods to establish quantitative relationships between predictors and outcomes, thereby individualizing the assessment of event probability [12]. Compared to traditional single-variable analyses, machine learning can handle high-dimensional data and capture non-linear interactions between variables. By integrating multiple algorithms, it can screen for more robust and clinically meaningful predictors [13,14]. Among them, a nomogram serves as a visual representation of a risk prediction model. By integrating the scores of multiple independent predictive indicators, it can intuitively predict individual outcome risks and has been widely used in oncology for prognosis judgment and complication risk prediction [15,16]. Compared to pure machine learning models that require complex computer knowledge, a nomogram only requires a ruler or simple calculations to derive a risk value, demonstrating better operability and scalability in clinical practice.

To date, most researchers have explored the influencing factors of illness uncertainty. A cross-sectional study on breast cancer patients found that social support levels and coping styles were significantly negatively correlated with illness uncertainty [17]. Another survey including 586 patients with lung cancer showed that anxiety, depression, and disease stage were independent predictors of illness uncertainty [18]. Additionally, demographic and clinical characteristics such as age, educational level, and treatment stage have also been confirmed to be associated with illness uncertainty [19,20]. However, although several systematic reviews and meta-analyses have synthesized these correlates, the majority of primary studies remain cross-sectional in design, focusing on correlation analyses of single or limited psychosocial variables; consequently, they do not provide predictive models that utilize multi-algorithm joint screening and can quantify individual risk. More critically, existing models severely lack consideration of the temporal dimension, such as differences in patient characteristics and treatment modes during different admission periods, and their stability and extrapolability have not been rigorously verified through time-stratified validation [21]. Because illness uncertainty is comprehensively affected by multi-dimensional factors such as a patient’s cognition, emotions, and social support, predictors screened by a single machine learning algorithm may have biases. Integrating multiple algorithms to screen core variables jointly can significantly improve the robustness and clinical interpretability of the predictors [22].

Therefore, this study uses the MUIS score as the outcome and employs LASSO, Random Forest, and XGBoost to jointly screen predictors. Each algorithm offers complementary strengths: LASSO shrinks irrelevant coefficients, Random Forest captures non-linear interactions, and XGBoost provides gradient boosted importance. Taking the intersection of variables selected by all three reduces overfitting risk from single algorithm choice. The final nomogram is deliberately based on logistic regression rather than a black box ensemble, ensuring transparent, bedside usable scoring while still benefiting from machine learning-guided variable preselection. We construct an individualized nomogram and evaluate it via internal and time-stratified validation, explicitly testing temporal stability, a dimension largely overlooked previously. The aim is a simple, objective tool for early risk stratification, potentially shifting uncertainty identification from post hoc assessment to admission screening.

## 2. Materials and Methods

### 2.1. Study Design

This was a single-center, cross-sectional study conducted at the Department of Oncology, Peking University People’s Hospital, Beijing, between August 2024 and January 2025. Patients were consecutively recruited from inpatient wards and outpatient clinics. Eligibility was assessed through a two-step process: initial screening of electronic medical records, followed by confirmation during routine consultations with the attending physician. The study was performed in accordance with the Declaration of Helsinki. The protocol was approved by the hospital’s Institutional Review Board (Ethics Approval Number: 2024PHB227-001), and written informed consent was obtained from all participants prior to enrollment.

### 2.2. Participants

Inclusion criteria were: (1) age ≥ 18 years; (2) histopathologically or cytologically confirmed diagnosis of a malignant tumor, all clinical stages, including locally advanced and metastatic disease were eligible, primary tumor types covered digestive system, respiratory system, gynecological, breast, hematologic, urologic, bone tumors, and other miscellaneous malignancies; (3) alert, oriented, with no communication barriers, and capable of independently completing self-administered questionnaires; (4) provision of written informed consent.

Exclusion criteria comprised: (1) admission to the intensive care unit during the study period; (2) terminal illness, operationally defined as an estimated life expectancy of less than 3 months or an Eastern Cooperative Oncology Group (ECOG) performance status of 4, as determined by the primary oncology team; (3) pre-existing severe psychiatric disorders (e.g., schizophrenia, bipolar disorder, or major depressive disorder with psychotic features) or documented dementia/cognitive impairment that could potentially confound the assessment of anxiety and depressive symptoms.

### 2.3. Sample Size

The sample size was planned to support subsequent prediction model development. With 18 candidate predictors, we adopted the rule of 10 events per predictor variable [23]. Based on an estimated prevalence of approximately 30% for high illness uncertainty in our target population, as suggested by previous literature and our local pilot data [2,18], the minimum required sample was calculated as 600 patients.

### 2.4. Data Collection

Data were collected using an electronic self-administered questionnaire created and distributed via the Wenjuanxing platform. A standardized instruction script was read to all participants, explaining the study purpose, confidentiality, and voluntariness, as well as basic operational steps. After providing written informed consent, patients scanned a QR code with their mobile phones to access the questionnaire. Researchers remained available to offer technical assistance only and did not interpret, paraphrase, or otherwise influence responses to any item, in order to minimize social desirability bias. For missing item-level data, we applied the following rule: if fewer than 10% of items on a given scale were missing, the missing values were imputed using the subscale mean; otherwise, the entire questionnaire was considered invalid and excluded.

Based on literature review and clinical experience [2,24], 18 candidate predictors were selected, covering three domains: (1) demographic characteristics (age, gender, education, residence, monthly household income, marital status, occupation, payment method, religious belief, and primary caregiver); (2) disease-related clinical features (disease duration, tumor type, smoking, and alcohol use); (3) psychosocial factors. The psychosocial instruments were administered in their validated Chinese versions, as follows:

Depression assessed using the 9-item Patient Health Questionnaire (PHQ-9) [25]. This study referred to the Chinese version adapted by Bian et al. [26]. Each item is scored from 0 to 3, with total scores ranging from 0 to 27. Scores of 0–4 indicate no depression, 5–9 mild, 10–14 moderate, 15–19 moderately severe, and 20–27 severe depression. In the present study, the Cronbach’s α for the PHQ-9 was 0.825.

Anxiety evaluated using the 7-item Generalized Anxiety Disorder Scale (GAD-7) [27]. The study used the version translated by He et al. [28]. Each item is scored from 0 to 3, with total scores ranging from 0 to 21. Scores of 0–4 indicate no anxiety, 5–9 mild, 10–14 moderate, and 15–21 severe anxiety. The GAD-7 showed a Cronbach’s α of 0.840 in this sample.

Medical coping measured using the Medical Coping Modes Questionnaire (MCMQ) [29]. This study referred to the Chinese version adapted by Shen et al. [30], which consists of 20 items across three subscales: confrontation, avoidance, and resignation. Each item is rated on a 1-to-4 Likert scale; higher subscale scores indicate a greater tendency to adopt that particular coping style. In the present study, the Cronbach’s α for the MCMQ was 0.901.

Social support assessed using the Social Support Rating Scale (SSRS). The SSRS was originally developed by Xiao et al. [31], which comprises 10 items covering three dimensions: objective support, subjective support, and utilization of support. Scoring follows the established manual, with higher total scores reflecting greater perceived social support. In the present study, the Cronbach’s α for the SSRS was 0.768.

### 2.5. Patient Illness Uncertainty Assessment and Grouping

The Mishel Uncertainty in Illness Scale (MUIS) was first developed by Mishel [32] in 1981. This study used a revised Chinese version that has undergone reliability and validity testing [7]. The scale consists of 33 items across 4 dimensions: ambiguity (13 items), complexity (7 items), lack of information (7 items), and unpredictability (5 items). Each item is scored on a 5-point Likert scale ranging from 1 (strongly disagree) to 5 (strongly agree). Notably, item 15 is not included in the total score; therefore, the effective total score ranges from 32 to 160. In the current sample, the Cronbach’s α coefficient for the total MUIS was 0.828, with subscale values ranging from 0.73 to 0.89, indicating good internal consistency.

Based on the score distribution, patients were initially classified into low (32–74.7), moderate (74.8–117.4), and high (117.5–160) uncertainty levels. Clinically, the moderate group represents a gray zone with considerable distress, yet these patients remain below the high uncertainty threshold. For a binary screening tool designed to identify those most in need of intensive intervention, combining the low and moderate groups into a single low-to-moderate risk category is pragmatic and consistent with prior studies that have used the upper tertile or mean plus one standard deviation as the cut-off for defining high risk [33]. Given the sample distribution characteristics and the need for balanced groups in the subsequent binary logistic regression analyses, the low and moderate groups were merged into a single “low-to-moderate illness uncertainty group,” while the high group was retained as the “high illness uncertainty group.” Accordingly, patients with MUIS scores ≤ 117.4 were assigned to the low-to-moderate group, and those with scores ≥ 117.5 were assigned to the high group.

### 2.6. Data Analysis

Data analysis was performed using R 4.5.1 software. All tests were two-sided, with a significance level of α = 0.05. Normally distributed quantitative data were expressed as mean ± standard deviation, and comparisons between groups were made using independent sample *t*-tests. Skewed data were expressed as M (P25, P75), and comparisons were made using the Mann–Whitney *U* test. Categorical data were described as frequencies (percentages) [*n* (%)], and comparisons were made using the *χ*^2^ test.

Three machine learning algorithms including LASSO regression, Random Forest, and XGBoost were used to screen for common predictors. All models were evaluated using 10-fold cross-validation. The RF model was trained using 500 decision trees, sorting variable importance using the Mean Decrease Gini index and retaining the top 10 most important variables, as their cumulative importance accounted for the majority of predictive contribution. For LASSO regression, non-informative variables were compressed to zero coefficients through a binomial penalty, using the minimum criteria and the 1-standard-error rule to select the optimal regularization parameter; variables with non-zero coefficients under the λ.1se criterion were considered significant predictors. In XGBoost, Shapley Additive Explanations (SHAP) were calculated to quantify each variable’s contribution to the model’s prediction, selecting the top 10 variables sorted by gain. Default tuning parameters were used for both RF and XGBoost models, as optimized through the 10-fold cross-validation procedure. Ultimately, the common variables identified by the three methods were visualized using a Venn diagram. Based on these common variables, a logistic regression model was built to construct the nomogram. The model’s discrimination was evaluated using the Receiver Operating Characteristic curve and Area Under the Curve, while consistency was assessed using calibration curves, followed by model validation.

## 3. Results

### 3.1. Patient Characteristics

Of the 1050 questionnaires distributed, 1006 were returned, and the response rate was 95.8%. After excluding 40 invalid responses, 966 valid questionnaires were retained for the final statistical analysis, yielding an effective response rate of 92.0%. Of these, 676 patients were assigned to the model development set and 290 to the time-stratified validation set. The development set was randomly divided into a training set (*n* = 474) and an internal validation set (*n* = 202) at a 7:3 ratio. Comparison of baseline data between the training and internal validation sets showed that, except for depression scores (*p* = 0.0321), all other variables were evenly distributed with no statistically significant differences (*p* > 0.05), indicating good random grouping and internal consistency within the development set. The clinical characteristics of the study sample are presented in Table 1. In the training set (*n* = 474), there were 328 cases (69.2%) in the high illness uncertainty group and 146 cases (30.8%) in the low-to-moderate group. Univariate analysis showed significant differences between the two groups in depression (*p* < 0.001), anxiety (*p* < 0.001), and social support (*p* < 0.001). The depression and anxiety scores in the high group were significantly higher than those in the low-to-moderate group, while social support scores were significantly lower, suggesting that psychological state and social support levels may be important influencing factors. Other variables such as age, gender, educational level, residence, disease duration, and diagnosis showed no statistically significant differences (*p* > 0.05) (Table 2).

### 3.2. Machine Learning Results

#### 3.2.1. LASSO Regression Preliminary Screening

LASSO regression selected anxiety as the only non-zero predictor under the λ.1se criterion.

#### 3.2.2. Random Forest Importance Ranking

**Figure 1:**
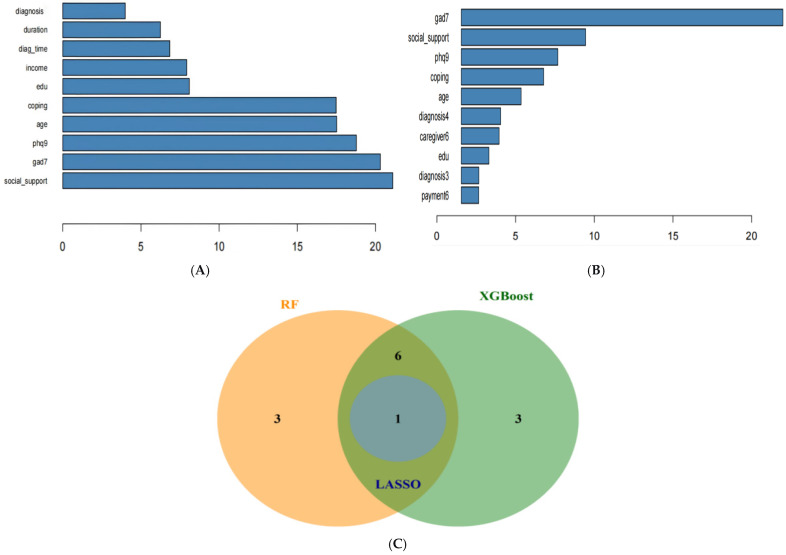
Core predictors identified by integrated machine learning algorithms. (**A**) Random Forest variable importance ranking. (**B**) XGBoost variable importance ranking. (**C**) Venn diagram of the three algorithms.

The Out-of-Bag error rate plot of the RF model showed a rapid decline as the number of decision trees increased, stabilizing after reaching about 200 trees. The error rate curves for both high and low-to-moderate groups also remained stable with no upward trend, indicating good model stability. By calculating the Mean Decrease Gini index, the top 10 important variables were: social support, anxiety, depression, age, medical coping modes, educational level, monthly household income, time since diagnosis, disease duration, and disease diagnosis (Figure 1A).

#### 3.2.3. XGBoost Importance Ranking

Variable importance ranking (Figure 1B) showed the top 10 variables were: anxiety, social support, depression, medical coping modes, age, disease diagnosis, caregiver, educational level, time since diagnosis, and medical payment method.

#### 3.2.4. Common Factor Screening

The three algorithms showed different degrees of overlap in variable selection. LASSO regression selected anxiety as the only non-zero predictor. Random Forest and XGBoost identified several overlapping predictors. The Venn diagram showed that anxiety was the only variable shared by all three algorithms. Using the predefined consensus rule of selection by at least two algorithms, seven predictors were retained for the final nomogram: age, education level, diagnosis, depression, anxiety, medical coping, and social support. Collinearity diagnostics showed that the Variance Inflation Factor (VIF) of each variable was less than 4, indicating no severe multicollinearity (Figure 1C). In predictive modeling, variables that are not statistically significant in univariate analysis can still contribute to multivariable prediction by capturing joint effects or interactions. Therefore, we retained all seven variables identified by the ensemble algorithms.

### 3.3. Nomogram Construction and Validation

**Figure 2:**
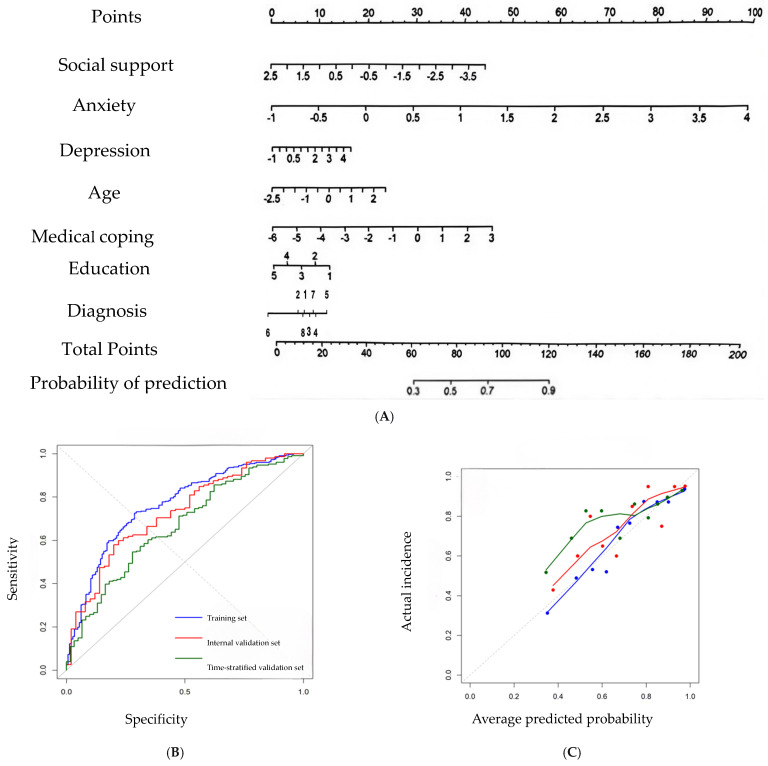
Construction and validation of the risk prediction nomogram. **Notes:** In the nomogram, education level is coded as 1 = primary school or below, 2 = junior high school, 3 = high school or junior college, 4 = bachelor’s degree, and 5 = master’s degree or above. Diagnosis is represented by the dummy variable diagnosis 3, where 1 indicates gynecological cancer and 0 indicates other cancer types. (**A**) Nomogram. (**B**) ROC curves. (**C**) Calibration curves.

The aforementioned seven variables were included in a logistic regression to build a nomogram model. Each factor corresponds to a specific score, and the total score is calculated by summing these seven scores and locating the sum on the total points axis. Because variables were standardized, the scales for continuous variables in the nomogram reflect standardized scores (Figure 2A). In practical application, patients’ raw values must first be converted into standardized values based on the training set’s mean and standard deviation before reading the individual scores and predicted probabilities on the nomogram (means and standard deviations in Table 3). Discrimination evaluation: the AUC for the training set was 0.763 (95% CI: 0.717–0.808), 0.724 for the internal validation set (95% CI: 0.645–0.803), and 0.663 for the time-stratified validation set (95% CI: 0.587–0.739) (Figure 2B). Calibration evaluation: both the training set (*χ*^2^ = 9.438, *p* = 0.307, Brier = 0.174) and the internal validation set (*χ*^2^ = 14.169, *p* = 0.078, Brier = 0.168) demonstrated good calibration, with highly consistent predicted and actual observed risks. In the time-stratified validation set, the HL test indicated calibration bias (*χ*^2^ = 29.831, *p* < 0.001, Brier = 0.177), and the calibration curve showed that the model underestimated the risk for low-to-moderate group patients in this population (Figure 2C). Full classification metrics and decision curve analysis are available in the Supplementary Materials.

## 4. Discussion

### 4.1. Illness Uncertainty Predictors

#### 4.1.1. The Core Role of Anxiety and Depression

In this study, anxiety and depression were strongly associated with illness uncertainty. Univariate analysis showed significantly higher scores in the high-risk group (*p* < 0.001). Notably, anxiety was the only variable jointly identified by all three algorithms (LASSO, RF, XGBoost), suggesting it may be a key psychological correlate rather than a causal driver [34]. This aligns closely with Mishel’s uncertainty in illness theory [35]: when patients are anxious, their information processing abilities may decline, potentially making them more prone to negative interpretations and catastrophic thinking regarding disease information, which could exacerbate uncertainty [36]. Depression is linked to negative cognitive biases, which may direct attention to negative cues and potentially amplify perceived uncertainty [37]. Studies suggest mood disorders are associated with altered appraisal processes, possibly contributing to confusion and worry [38]. Both anxiety and depression are modifiable factors; early referral to psycho-oncology or interventions may improve mood and might indirectly reduce uncertainty.

#### 4.1.2. The Protective Role of Social Support and Coping Styles

Social support showed significant group differences (high group 41.0 vs. low-to-moderate group 46.0, *p* < 0.001) and was identified by both RF and XGBoost as a key predictor. As an external resource, its protective effects operate on multiple levels: emotional support alleviates fear, informational support aids understanding, and tangible support reduces life burdens [7]. Previous studies confirm that social support is negatively correlated with uncertainty and acts as a crucial protective factor for psychological adaptation [24]. Furthermore, stress coping theory indicates individuals adopt different strategies when facing stress [39]. In this model, medical coping styles were included, suggesting patients leaning towards “confrontation” strategies exhibit lower uncertainty, while those using “avoidance” or “resignation” show higher levels. This matches Lazarus’ stress coping theory [40]. Research highlights an interaction between social support and coping: high support provides resources that foster positive coping, while positive coping helps patients better utilize available support [41]. This virtuous cycle appears to be an important pathway associated with reduced uncertainty.

#### 4.1.3. The Impact of Demographic and Clinical Characteristics

Age, educational level, and disease diagnosis were identified as predictors. Older patients may experience declines in information processing and cognitive flexibility, struggling with complex medical information, which aligns with Wang, T. et al.’s findings [42]. Patients with lower educational levels have limited ability to acquire and comprehend medical information. This implies that communication with older or less-educated patients should use plain language, with increased repetition and confirmation [43]. Disease diagnosis reflects the differing psychological impacts of various tumors, which possess unique trajectories, treatment complexities, and prognoses [44]. For instance, patients with better prognoses might focus on recurrence risks and long-term quality of life, whereas those with poorer prognoses focus on survival time and symptom control. This highlights the need for specialized, individualized psychological assessments and support tailored to specific diagnostic types.

### 4.2. Model Predictive Performance and Validation

The constructed nomogram demonstrated good discrimination in the training set (AUC = 0.763) and internal validation set (AUC = 0.724). Calibration tests showed high consistency between predicted and actual risks (*p* > 0.05), indicating stable predictive performance within the development and concurrent random populations. Compared to previous studies, this model’s predictive efficacy is modest. In the stricter time-stratified validation, discrimination decreased (AUC = 0.663), and significant calibration bias emerged (*p* < 0.001), primarily underestimating risk in low-to-moderate risk individuals. This phenomenon is similar to the “temporal drift” observed by Bevilacqua et al. [45] in building a BCRL predictive model. This may be due to subtle shifts over time in patient characteristics, treatment modalities, or nursing focuses at our center. For example, changes in patients’ disease awareness, access to medical information, and psychosocial resources over time can alter uncertainty mechanisms [9,46]. Brier score (0.177) remains acceptable, but calibration bias urges caution. Notably, the time-stratified cohort came from the same institution but a later window, showing that even single-center temporal heterogeneity can degrade performance. This implies that cross-sectionally developed models may have limited time transportability; routine updating or recalibration may be needed for longitudinal use. Independent multi-center validation with longer follow-up is essential to assess whether drift is random or systematic, and this temporal decay should be given greater weight when considering the model’s readiness for practice.

### 4.3. Strengths and Limitations

This is one of the few studies integrating three machine learning algorithms, LASSO regression, Random Forest and XGBoost, to jointly screen the predictors of illness uncertainty in patients with malignant tumors, so as to enhance the robustness and clinical interpretability of the selected variables. In addition, time-stratified validation was used to evaluate the stability of the model in patients at different time points of diagnosis, which was more rigorous than traditional internal validation. The final nomogram included seven predictors, including potentially modifiable psychological and social factors, such as anxiety, depression, social support, and coping style, which provided a clear target for clinical psychological intervention. At the same time, its visual form is helpful for early risk stratification and doctor–patient communication.

However, several limitations should be acknowledged. The single-center cross-sectional design may have introduced selection bias and limits generalizability, and the temporal ordering of predictors and outcome cannot be established. Thus, the model effectively classifies patients according to their current profiles rather than predicting future psychological states. The dichotomization of MUIS scores into high versus low-to-moderate risk may have lost information, and the chosen cut-off of 117.5 lacks established clinical validation. The time-stratified validation, while more stringent than internal validation, still originates from the same institution and does not constitute independent external validation from another center, limiting transportability. All psychosocial predictors were derived from self-reported questionnaires, subject to recall and social desirability bias. The model’s overall discriminative performance with an internal AUC of 0.724 is only moderate, and the calibration deviation in the time-stratified set (*p* < 0.05) indicates temporal heterogeneity and poor transportability over time, necessitating caution in clinical application. Therefore, the model should be used only as a preliminary screening aid, not as a definitive diagnostic tool. Future multi-center prospective studies are needed to further validate and optimize this model, and subsequent research could consider constructing dynamic prediction models that capture longitudinal changes in illness uncertainty.

### 4.4. Implications for Practice

Despite its limitations, this model offers potential clinical value as a preliminary screening tool. It shifts the identification window for illness uncertainty from “post hoc assessment” to “initial admission screening,” providing a quantitative basis for stratified psychological nursing, although the approach still requires collection of several standardized questionnaires. By collecting seven routine variables upon admission, medical staff can rapidly conduct risk assessments, medical staff can rapidly conduct risk assessments, transitioning from “passive response” to “proactive screening. Secondly, among the seven predictors, anxiety, depression, social support, and coping modes are modifiable, offering clear intervention targets: (1) for patients with high anxiety/depression scores, early referrals to psycho-oncology or pharmacological interventions may be beneficial, and clinical decisions should consider individual patient circumstances; (2) for those with low social support, family meetings and peer support groups can strengthen social resource links; (3) for patients with negative coping styles, health education can help build positive disease-coping strategies. Finally, the nomogram’s visual nature makes it an effective auxiliary tool for doctor–patient communication. Staff can intuitively display risk probabilities and explain related factors to patients, enhancing their understanding and acceptance of their psychological state and improving compliance with subsequent psychological interventions.

## 5. Conclusions

Based on three machine learning algorithms, this study constructed and internally validated a nomogram for concurrent risk stratification of illness uncertainty in patients with malignant tumors. Seven core predictors were identified: age, education level, disease diagnosis, depression, anxiety, medical coping style, and social support. Anxiety, being the only variable selected by all three algorithms, suggests it may be a prominent psychological correlate of illness uncertainty rather than a causal mechanism. The model showed good discrimination in the training and internal validation sets, but calibration bias was observed in the time-stratified validation set, indicating temporal heterogeneity. Given the single-center cross-sectional design, dichotomization of MUIS, lack of multi-center temporal validation, and self-report biases, this nomogram should be viewed as an exploratory tool that requires further external validation and optimization before any clinical implementation can be considered. Modifiable predictors provide clear targets for psychosocial interventions. Future multi-center prospective studies are needed to further refine and externally validate the model.

## Figures and Tables

**Table 1:** Characteristics of the participants in the training and internal validation sets (*n* = 676).

Variable	Training Set (*n*)	Internal Validation Set (*n* = 202)	*χ*^2^/*Z*	*p* Value
**Age** M[P_25_,P_75_]	50.00 (36.2, 61.0)	47 (38.0, 59.0)	0.477	0.634
**Gender** *n* (%)			0.704	0.401
Male	345 (72.8)	154 (76.2)		
Female	129 (27.2)	48 (23.8)		
**Education** *n* (%)			1.743	0.783
Primary school or below	44 (9.3)	23 (11.4)		
Junior high school	103 (21.7)	49 (24.3)		
High school or junior college	170 (35.9)	65 (32.2)		
Bachelor’s degree	131 (27.6)	53 (26.2)		
Master’s degree or above	26 (5.5)	12 (5.9)		
**Residence** *n* (%)			2.131	0.775
Rural	126 (26.6)	54 (26.7)		
Town	118 (24.9)	53 (26.2)		
Urban	230 (48.5)	95 (47.0)		
**Monthly household income per capita** *n* (%)			2.886	0.577
≤2000 RMB	91 (19.2)	35 (17.3)		
2001–5000 RMB	139 (29.3)	51 (25.2)		
5001–10,000 RMB	123 (25.9)	55 (27.2)		
10,001–20,000 RMB	78 (16.5)	36 (17.8)		
>20,000 RMB	43 (9.1)	25 (12.4)		
**Marital status** *n* (%)			5.306	0.169
Unmarried	77 (16.2)	29 (14.4)		
Married	358 (75.5)	162 (80.2)		
Divorced	23 (4.9)	3 (1.5)		
Widowed	16 (3.4)	8 (4.0)		
**Occupation** *n* (%)			3.298	0.115
Manual worker	113 (23.9)	43 (21.3)		
Mental worker	65 (13.7)	23 (11.3)		
Retired	118 (24.9)	47 (23.3)		
Others	178 (37.6)	89 (44.1)		
**Method of medical payment** *n* (%)			3.201	0.798
Urban employee medical insurance	322 (67.9)	147 (72.8)		
New rural cooperative medical scheme	119 (25.1)	41 (20.3)		
Government-funded medical care	11 (2.3)	4 (2.0)		
Self-pay	11 (2.3)	6 (3.0)		
Commercial insurance	4 (0.8)	1 (0.5)		
Others	7 (1.5)	3 (1.5)		
**Religious belief** *n* (%)			0.187	0.665
Yes	455 (96.0)	196 (97.0)		
No	19 (4.0)	6 (3.0)		
**Primary caregiver** *n* (%)			7.943	0.159
Spouse	207 (43.7)	99 (49.0)		
Parent	84 (17.7)	34 (16.8)		
Child	111 (23.4)	38 (18.8)		
Sibling	24 (5.1)	6 (3.0)		
Nurse	26 (5.5)	19 (9.4)		
Others	22 (4.6)	6 (3.0)		
**Disease duration** *n* (%)			1.884	0.596
<1 month	147 (31.0)	67 (33.2)		
2–5 months	177 (37.3)	78 (38.6)		
6–12 months	64 (13.5)	29 (14.4)		
>12 months	86 (18.1)	28 (13.9)		
**Cancer type** *n* (%)			0.987	0.117
Digestive system cancer	43 (9.1)	22 (11.1)		
Respiratory system cancer	2 (0.4)	2 (1.0)		
Gynecological cancer	107 (22.6)	51 (25.2)		
Breast cancer	140 (29.5)	70 (34.7)		
Hematologic malignancy	13 (2.7)	8 (4.0)		
Urologic cancer	2 (0.4)	1 (0.5)		
Bone tumor	146 (30.8)	43 (21.3)		
Others	21 (4.4)	5 (2.5)		
**Smoking history** *n* (%)			0.291	0.589
Yes	80 (16.9)	30 (14.9)		
No	394 (83.1)	172 (85.1)		
**Alcohol consumption** *n* (%)			0.251	0.616
Yes	89 (18.8)	42 (20.8)		
No	385 (81.2)	160 (79.2)		
**Depression** M[P_25_,P_75_]	3.00 (0.00, 7.00)	4.00 (1.00, 8.70)	2.147	0.032
**Anxiety** M[P_25_,P_75_]	2.00 (0.00, 7.00)	2.00 (0.00, 7.00)	0.369	0.712
**Medical coping** M[P_25_,P_75_]	52.00 (49.00, 54.00)	51.00 (49.00, 54.70)	0.094	0.925
**Social support** M[P_25_,P_75_]	43.00 (37.00, 48.00)	43.00 (37.00, 49.00)	0.281	0.779

**Notes:** Data are presented as median (interquartile range, IQR) for continuous variables and number (%) for categorical variables. Group comparisons were performed using the Mann–Whitney *U* test (*Z*) for continuous variables and the chi-square test (*χ*^2^) for categorical variables. Abbreviations: RMB, Renminbi (Chinese Yuan; 1 USD ≈ 7.2 RMB at the time of data collection). Occupational categories were defined as follows: “manual worker” included individuals engaged in blue collar occupations, agricultural production, and service industry personnel; “mental worker” included professionals such as teachers, physicians, lawyers, and government/enterprise administrative staff; “retired” referred to individuals who had formally withdrawn from the workforce due to age or other reasons; “others” included students, military personnel, unemployed individuals, and those not classifiable into the above categories.

**Table 2:** Comparison of characteristics between the high and low-to-moderate illness uncertainty groups in training set.

Variable	High Illness Uncertainty Group (*n* = 328)	Low-to-Moderate Illness Uncertainty Group (*n* = 146)	*χ*^2^/*Z*	*p* Value
**Age** M[P_25_,P_75_]	50.00 (39.00, 61.00)	49.00 (34.25, 60.75)	2.572	0.198
**Gender** *n* (%)			0.156	0.693
Male	241 (73.5)	104 (71.2)		
Female	87 (26.5)	42 (28.8)		
**Education** *n* (%)			6.134	0.189
Primary school or below	32 (9.8)	12 (8.2)		
Junior high school	74 (22.6)	29 (19.9)		
High school or junior college	114 (34.8)	56 (38.4)		
Bachelor’s degree	95 (29.0)	36 (24.7)		
Master’s degree or above	13 (4.0)	13 (8.9)		
**Residence** *n* (%)			4.801	0.063
Rural	93 (28.4)	33 (22.6)		
Town	71 (21.6)	47 (32.2)		
Urban	164 (50.0)	66 (45.2)		
**Monthly household income per capita** *n* (%)			6.209	0.184
≤2000 RMB	68 (20.7)	23 (15.8)		
2001–5000 RMB	101 (30.8)	38 (26.0)		
5001–10,000 RMB	79 (24.1)	44 (30.1)		
10,001–20,000 RMB	48 (14.6)	30 (20.5)		
>20,000 RMB	32 (9.8)	11 (7.5)		
**Marital status** *n* (%)			5.013	0.953
Unmarried	53 (16.2)	24 (16.4)		
Married	248 (75.6)	110 (75.3)		
Divorced	15 (4.6)	8 (5.5)		
Widowed	12 (3.7)	4 (2.7)		
**Occupation** *n* (%)			6.661	0.753
Manual worker	76 (23.1)	37 (25.4)		
Mental worker	47 (14.3)	18 (12.3)		
Retired	83 (25.3)	35 (24.0)		
Others	122 (37.2)	56 (38.4)		
**Method of medical payment** *n* (%)			7.801	0.056
Urban employee medical insurance	221 (67.4)	101 (69.2)		
New rural cooperative medical scheme	86 (26.2)	33 (22.6)		
Government-funded medical care	7 (2.1)	4 (2.7)		
Self-pay	10 (3.0)	1 (0.7)		
Commercial insurance	3 (0.9)	1 (0.7)		
Others	1 (0.3)	6 (4.1)		
**Religious belief** *n* (%)			0.032	0.858
Yes	314 (95.7)	141 (96.6)		
No	14 (4.3)	5 (3.4)		
**Primary caregiver** *n* (%)			6.840	0.232
Spouse	145 (44.2)	62 (42.5)		
Parent	58 (17.7)	26 (17.8)		
Child	81 (24.7)	30 (20.5)		
Sibling	17 (5.2)	7 (4.8)		
Nurse	17 (5.2)	9 (6.2)		
Others	10 (3.0)	12 (8.2)		
**Disease duration** *n* (%)			0.266	0.966
<1 month	101 (30.8)	46 (31.5)		
2–5 months	123 (37.5)	54 (37.0)		
6–12 months	43 (13.1)	21 (14.4)		
>12 months	61 (18.6)	25 (17.1)		
**Cancer type** *n* (%)			7.891	0.084
Digestive system cancer	30 (9.1)	13 (8.9)		
Respiratory system cancer	1 (0.3)	1 (0.7)		
Gynecological cancer	66 (20.1)	41 (28.1)		
Breast cancer	107 (32.6)	33 (22.6)		
Hematologic malignancy	11 (3.4)	2 (1.4)		
Urologic cancer	1 (0.3)	1 (0.7)		
Bone tumor	101 (30.8)	45 (30.8)		
Others	11 (3.4)	10 (6.8)		
**Smoking history** *n* (%)			0.039	0.981
Yes	53 (16.8)	25 (17.1)		
No	273 (83.2)	121 (82.9)		
**Alcohol consumption** *n* (%)			0.283	0.595
Yes	59 (18.0)	30 (20.5)		
No	269 (82.0)	116 (79.5)		
**Depression** M[P_25_,P_75_]	4.50 (1.00, 8.25)	1.00 (0.00, 4.00)	3.340	<0.001
**Anxiety** M[P_25_,P_75_]	4.00 (0.00, 7.00)	2.00 (1.00, 4.00)	3.386	<0.001
**Medical coping** M[P_25_,P_75_]	52.00 (49.00, 54.00)	52.00 (49.00, 55.00)	2.289	0.445
**Social support** M[P_25_,P_75_]	41.00 (37.00, 47.00)	46.00 (41.00, 50.00)	6.882	<0.001

**Notes:** Data are presented as median (interquartile range, IQR) for continuous variables and number (%) for categorical variables. Group comparisons were performed using the Mann–Whitney *U* test (*Z*) for continuous variables and the chi-square test (*χ*^2^) for categorical variables.

**Table 3:** Coding and distribution of predictors included in the nomogram in the training set.

Predictor	Variable Type/Coding	Training Set Value
Age	Continuous, years	52.3 ± 15.2
Anxiety	Continuous, GAD-7	6.5 ± 5.0
Depression	Continuous, PHQ-9	5.9 ± 5.2
Medical coping	Continuous score	51.8 ± 4.9
Social support	Continuous score	43.2 ± 8.1
**Education level**	**Ordinal categorical**	
Primary school or below	Code = 1	44 (9.3%)
Junior high school	Code = 2	103 (21.7%)
High school or junior college	Code = 3	170 (35.9%)
Bachelor’s degree code	Code = 4	131 (27.6%)
Master’s degree or above	Code = 5	26 (5.5%)
**Diagnosis 3**	**Binary dummy variable**	
Gynecological cancer	Code = 1	107 (22.6%)
Other cancer types	Code = 2	367 (77.4%)

**Notes:** Continuous variables are presented as mean ± standard deviation. Categorical variables are presented as number and percentage. Education level was entered as an ordinal categorical variable coded from 1 to 5. Diagnosis was entered as a binary dummy variable, diagnosis 3, where 1 indicates gynecological cancer and 0 indicates all other cancer types. Continuous predictors were standardized when constructing the nomogram using the mean and standard deviation from the training set.

## Data Availability

Due to patient privacy restrictions, the data are not publicly available. De-identified data can be requested from the corresponding author.

## Supplementary Materials

The following supporting information can be downloaded at: https://www.mdpi.com/article/10.3390/healthcare14142160/s1, Table S1. Discrimination, calibration, and classification performance of the nomogram. Supplementary File S1: Informed Consent.

## Abbreviations

The following abbreviations are used in this manuscript:

AUC	Area Under the Curve
BCRL	Breast Cancer-Related Lymphedema
CBT	Cognitive-Behavioral Therapy
CI	Confidence Interval
HL	Hosmer–Lemeshow
LASSO	Least Absolute Shrinkage and Selection Operator
MUIS	Mishel Uncertainty in Illness Scale
RF	Random Forest
ROC	Receiver Operating Characteristic
SHAP	Shapley Additive Explanations
VIF	Variance Inflation Factor
XGBoost	eXtreme Gradient Boosting
