# Supplementary material for: Development and Validation of a Machine Learning-Based Prediction Model for Illness Uncertainty in Patients with Malignant Tumors
Source: Healthcare (Basel). 2026 Jul 17;14(14):2160. doi: 10.3390/healthcare14142160 (PMC13410291; doi:10.3390/healthcare14142160)
Supplement: Supplementary file 1 [file healthcare-14-02160-s001.zip › Ethical Approval Document (billingual)).pdf]

北京大学人民医院伦理审查委员会
伦理审查批件

批　件　号	2024PHB227-001		
项目名称	恶性肿瘤患者疾病不确定感对抑郁症状的纵向影响：应对方式的中介作用和社会支持的调节作用		
项目来源	自选课题		
研究单位	北京大学人民医院	承担科室	妇产科
主要研究者	李晓丹	职　称	主任护师

审查类别	审查方式	审查日期
初始审查	快速审查	2024/06/20

审查地点	北京市西城区西直门南大街11号　北京大学人民医院伦理审查委员会
审查委员	昌晓红
审批文件	1.初始审查申请：2024/06/19<br>2.研究方案：1.0,2024/05/13<br>3.知情同意书：1.0,2024/05/13<br>4.科学性审查批件<br>5.主要研究者履历

Ethical Review Approval from the Ethics Review Committee of Peking University People's Hospital

Batch ID	2024PHB227-001		
Project name	Longitudinal effect of disease uncertainty on depressive symptoms in patients with malignant tumors: mediating role of coping style and moderating role of social support		
Project source	Custom topic		
Research Unit	Peking University People's Hospital	Department	Gynaecology and obstetrics
Principal Investigator	Li Xiaodan	Professional ranks and titles	Chief superintendent nurse

Review category	Review method	Review date
Initial review	Quick Review	2024/06/20

Review location	Ethics Review Committee, Peking University People's Hospital, No.11, Xizhimen South Street, Xicheng District, Beijing, China
Juror	Chang Xiaohong
Approval document	1. Initial review request: 2024/06/19<br>2. Research proposal: 1.0, 2024/05/13<br>3. Informed Consent Form: 1.0, 2024/05/13<br>4. Scientific review approval<br>5. Curriculum Vitae of Principal Investigator

审查意见：同意

根据《涉及人的生物医学研究伦理审查办法(2016)》、《药物临床试验伦理审查工作指导原则(2010)》、《赫尔辛基宣言》、《人体生物医学研究国际道德指南》,《中华人民共和国数据安全法》、《中华人民共和国个人信息保护法》、《中华人民共和国人类遗传资源管理条例》及其它相关法律、法规和政策，经本伦理审查委员会审查，同意按所批准的文件开展本项研究。

请遵循伦理审查委员会批准的方案开展研究，保护受试者的健康与权利。

研究过程中若变更主要研究者，对研究方案、知情同意书、招募材料等的任何修改，请申请人提交修正案审查申请。

申请人计划暂停或提前终止研究，请及时提交暂停/终止研究报告。

研究完成前，请申请人提交研究完成报告。

批件有效期	1年；截止日期：2025年06月20日<br>(如研究逾期未实施，需提出延长有效期申请)		
伦理审查委员会联系人	丛翠翠	联 系 电 话	010-88324516
伦理审查委员会	北京大学人民医院伦理审查委员会(盖章)		
主任委员签字	徐		
日　　　期	2024年06月21日		

Review comments: Agree

In accordance with the "Measures for the Ethical Review of Biomedical Research Involving Human Subjects (2016)", "Guidelines for Ethical Review of Clinical Drug Trials (2010)", "Helsinki Declaration", "International Ethical Guidelines for Biomedical Research Involving Human Subjects", "Data Security Law of the People's Republic of China", "Personal Information Protection Law of the People's Republic of China", "Regulations on Human Genetic Resources of the People's Republic of China", and other relevant laws, regulations, and policies, this Ethics Review Committee has reviewed and approved the implementation of this research project in compliance with the approved documents.

The study shall be conducted in accordance with the protocol approved by the Ethics Review Committee to protect the health and rights of the subjects.

If the principal investigator is changed during the research, and any modification is made to the research plan, informed consent form, recruitment materials, etc., the applicant shall submit an amendment application for review.

The applicant plans to suspend or terminate the study early. Please submit the suspension/termination report in time.

Before the study is completed, the applicant is requested to submit a study completion report.

Approval validity period	1 year; deadline: June 20,2025<br>(If the research is not implemented within the deadline, an application for extension of validity period is required)		
Ethics Committee contact	Cong Cuicu[illegible]	Contact number	010-88324516
Ethics Committee	Ethics Review Committee of Peking University People's Hospital (Seal)		
Signature of Chairperson	Xu		
Date	June 21,2024		
