# Supplementary material for: Development and Validation of a Machine Learning-Based Prediction Model for Illness Uncertainty in Patients with Malignant Tumors
Source: Healthcare (Basel). 2026 Jul 17;14(14):2160. doi: 10.3390/healthcare14142160 (PMC13410291; doi:10.3390/healthcare14142160)
Supplement: Supplementary file 1 [file healthcare-14-02160-s001.zip › File S1. Informed Consent Form.pdf]

Informed Consent

Shalom ！

We are conducting a study on the impact of disease uncertainty on depressive symptoms in patients with malignant tumors. As you have a tumor and your specific condition meets the enrollment criteria for this study, we would like to invite you to participate in this research.

This informed consent form will provide you with information regarding the purpose, procedures, benefits, risks, and inconveniences of this study, as well as your rights. Please read it carefully and make a well-informed decision about whether to participate in the study. When the investigator explains and discusses the informed consent form with you, you may ask questions at any time and request clarification on any points that are unclear to you. You may also consult with your family, friends, and your attending physician before making a decision.

If you are currently participating in another clinical study, please be sure to inform your study physician or investigator.

The principal investigator of this study is Li Xiaodan from Peking University People's Hospital, and the research funding or financial support was self-selected.

1. Why was this study conducted?

Malignant tumors are currently one of the leading causes of death worldwide, ranking second among global mortality factors. They have become a serious threat to the physical and mental health of residents in China. It is essential to focus on the psychological characteristics of this population, including understanding the disease uncertainty, depression, coping strategies, and social support status among malignant tumor patients. The study aims to analyze the influencing factors of disease uncertainty and explore the correlations between coping strategies among malignant tumor patients. This research provides a reference for healthcare professionals to better support malignant tumor patients, helping them adopt positive and effective coping strategies. Consequently, it seeks to reduce disease uncertainty, decrease depression incidence, promote physical and mental health, and ultimately improve the quality of life for malignant tumor patients.

2. Who will be invited to participate in this study?

The patients with confirmed malignant tumors in obstetrics and gynecology and breast surgery were the research objects.

3. How many people will participate in this study?

A total of 400 subjects were planned to be enrolled in this study.

4. How was the study conducted?

In this observational study, we administered questionnaires to hospitalized patients with malignant tumors using the General Condition Survey, 9-item Patient Health Questionnaire (PHQ-9), Generalized Anxiety Disorder Scale (GAD-7), Medical Uncertainty Scale (MUIS-A), and Social Support Scale. Investigators provided clear explanations to ensure patient comprehension, and the questionnaires were collected immediately after completion.

5. How long will this study last?

No observation follow-up was required in this study.

6. What are the risks of participating in this study?

This study is an observational study and will not pose any risks to your examination or treatment.

You will be asked to complete the survey at different stages, each taking approximately 20 minutes. Some questions may cause discomfort, and you may decline to answer them. We will protect your privacy as much as possible. This survey is for this specific study only and will not result in information leakage or misuse.

7. What are the benefits of participating in this study?

Your participation in this study will not yield immediate direct benefits, but your involvement will contribute to our understanding of disease uncertainty, depressive conditions, coping strategies, and social support status among patients with malignant tumors. It will also help analyze the influencing factors of disease uncertainty, providing references for healthcare professionals to better offer relevant support to these patients. This will assist them in adopting positive and effective coping strategies, thereby reducing disease uncertainty, promoting their physical and mental health, and ultimately improving the quality of life for patients with malignant tumors.

8. Is participation and completion of this study mandatory?

Your participation in this study is entirely voluntary. If you do not wish to participate, you may decline, and this will not have any negative impact on your current or future medical care. Your physician will continue to provide standard medical treatment as usual. Even if you agree to participate, you may change your mind at any time and inform the investigators to withdraw from the study. Your withdrawal will not affect your access to standard medical services. In principle, after your withdrawal, the investigators will strictly retain all collected information about you until final disposal, during which no further use or disclosure of such information will occur. Throughout the study, we will promptly inform you of any information that may influence your decision to continue participating in the study.

9. What are the costs and compensation for participating in the study?

This study is an observational study and will not incur additional examination fees. Questionnaires will be completed during hospitalization and follow-up visits, which will not consume excessive time from you, and no compensation will be provided.

10. Were participants in this study compensated?

This study was conducted on a voluntary basis, with no additional compensation provided to participants.

11. Management of research-related injuries?

This study is an observational study without any invasive procedures related to the research, and it will not cause physical harm. If any psychological discomfort occurs during the questionnaire completion, participants may discontinue participation at any time.

12. Is my information confidential?

If you decide to participate in this study, your participation and personal data will be kept confidential. Without your consent, no information that could identify you will be disclosed to any members outside the research team. All study participants and relevant parties will maintain your identity confidentiality as required. Your records will be stored in a locked filing cabinet, accessible only to researchers. When

necessary, government authorities or members of the ethics review committee may review your personal data at the research site in accordance with regulations. When the study results are published, no identifying information about you will be disclosed.

13. Who should I contact if I have questions or difficulties?

If you have any questions related to this study, please contact the investigator Li Xiaodan at 15010305099, available 24 hours a day.

For inquiries regarding your rights and interests, please contact the Ethics Review Committee of Peking University People's Hospital at 010-88324516.

Informed Consent Form (Signature Page)

Statement of the Investigator

I have informed the subject about the research background, purpose, procedures, risks, and benefits of the study on the uncertainty of disease in patients with malignant tumors, provided her with sufficient time to read the informed consent form, discuss with others, and addressed her questions regarding the study. I have informed the subject that she may contact Li Xiaodan at any time if she encounters issues related to the study, and that she may contact the Ethics Review Committee of Peking University People's Hospital at any time if she has questions regarding her rights/interests, and provided accurate contact information. I have informed the subject that she may withdraw from the study without any reason. I have informed the subject that she will receive a copy of the informed consent form, which includes both my signature and hers.

__________________ _________________

Signature of the investigator obtaining informed consent (regular script) Date

Signature of the investigator obtaining informed consent (regular script) Date

__________________ _________________

Signature of the investigator obtaining informed consent (handwritten) Date

Signature of the investigator obtaining informed consent (handwritten) Date

Subject Statement

I have been informed about the research background, objectives, procedures, risks, and benefits of the study on the uncertainty of disease in patients with malignant tumors and depression. I have had sufficient time and opportunities to ask questions, and I am satisfied with the responses to my inquiries. I have also been informed of whom to contact when I have questions, wish to report difficulties, concerns, provide suggestions for the study, seek further information, or offer assistance to the study. I have read this informed consent form and agree to participate in the study. I am aware that I may withdraw from the study at any time during the study period without any reason. I have been informed that I will receive a copy of this informed consent form, which includes my signature and the researcher's signature.

__________________ _________________

Subject's signature (regular script) Date Subject's signature (regular script) Date

__________________ _________________

Subject's signature (handwritten) Date Subject's signature (handwritten) Date

(When the subject lacks or is insufficient in the capacity for informed consent, the following methods shall be added or replaced)

___________________ _________________

Signature of legal representative (regular script) Date Signature of legal representative (regular script) Date

Signature of legal representative (handwritten) Date Signature of legal representative (handwritten) Date

Subject Relationship

Subject signature (if available) Date Subject signature (if available) Date
