# Supplementary material for: Development and Validation of a Machine Learning-Based Prediction Model for Illness Uncertainty in Patients with Malignant Tumors
Source: Healthcare (Basel). 2026 Jul 17;14(14):2160. doi: 10.3390/healthcare14142160 (PMC13410291; doi:10.3390/healthcare14142160)
Supplement: Supplementary file 1 [file healthcare-14-02160-s001.zip › Supplementary Table.pdf]

Table S1. Discrimination, calibration, and classification performance of the nomogram.

| Dataset | AUC (95% CI) | HLP | Brier | Calibration intercept | Calibration slope | Threshold | Sensitivity | Specificity | PPV | NPV | Accuracy |
|---|---|---|---|---|---|---|---|---|---|---|---|
| Training set | 0.763 (0.717–0.808) | 0.307 | 0.174 | 0.000 | 1.000 | 0.647 | 0.726 | 0.712 | 0.850 | 0.536 | 0.722 |
| Internal validation set | 0.724 (0.645–0.803) | 0.078 | 0.168 | 0.432 | 0.796 | 0.647 | 0.658 | 0.660 | 0.855 | 0.388 | 0.658 |
| Time-stratified validation set | 0.663 (0.587–0.739) | <0.001 | 0.177 | 0.867 | 0.552 | 0.647 | 0.629 | 0.557 | 0.842 | 0.286 | 0.614 |

Notes: HL, Hosmer–Lemeshow test; PPV, positive predictive value; NPV, negative predictive value. The classification threshold was determined in the training set by maximizing the Youden index and was then fixed at 0.647 for internal and time-stratified validation.
